# Humpback whales feed on hatchery-released juvenile salmon

**DOI:** 10.1098/rsos.170180

**Published:** 2017-07-12

**Authors:** Ellen M. Chenoweth, Janice M. Straley, Megan V. McPhee, Shannon Atkinson, Steve Reifenstuhl

**Affiliations:** 1 College of Fisheries and Ocean Sciences, University of Alaska Fairbanks, Juneau, AK 99801, USA; 2 Department of Natural Sciences, University of Alaska Southeast, Sitka, AK 99835, USA; 3 Northern Southeast Regional Aquaculture Association, Sitka, AK 99835, USA

**Keywords:** *Megaptera novaeangliae*, *Oncorhynchus* spp*.*, marine mammal–fishery interactions, foraging, aquaculture, behaviour

## Abstract

Humpback whales are remarkable for the behavioural plasticity of their feeding tactics and the diversity of their diets. Within the last decade at hatchery release sites in Southeast Alaska, humpback whales have begun exploiting juvenile salmon, a previously undocumented prey. The anthropogenic source of these salmon and their important contribution to local fisheries makes the emergence of humpback whale predation a concern for the Southeast Alaska economy. Here, we describe the frequency of observing humpback whales, examine the role of temporal and spatial variables affecting the probability of sighting humpback whales and describe prey capture behaviours at five hatchery release sites. We coordinated twice-daily 15 min observations during the spring release seasons 2010–2015. Using logistic regression, we determined that the probability of occurrence of humpback whales increased after releases began and decreased after releases concluded. The probability of whale occurrence varied among release sites but did not increase significantly over the 6 year study period. Whales were reported to be feeding on juvenile chum, Chinook and coho salmon, with photographic and video records of whales feeding on coho salmon. The ability to adapt to new prey sources may be key to sustaining their population in a changing ocean.

## 1. Background

Humpback whales (*Megaptera novaeangliae*) are notable among baleen whales for their diet diversity. Their large flukes and long pectoral fins allow for quick acceleration and manoeuvring, enabling humpback whales to capture highly mobile prey [1]. This energetically demanding filter feeding requires prey to be aggregated for capture by humpback whales [2,3]. Humpback whales demonstrate particularly complex and sometimes innovative foraging tactics [4–7]. Behavioural plasticity may be an important aspect of their persistence by allowing them to adapt to changing environments and avoid competition [8,9].

Humpback whales feed primarily on euphausiids and small schooling fish [10–12]. In Southeast Alaska, the humpback whale population has been increasing since the end of commercial whaling in the early 1970s [13–15]. Increased intraspecific competition can lead to an increase in a population's diet diversity with the inclusion of less-preferred prey items [9]. Humpback whales have not been documented feeding on wild juvenile salmon (Salmonidae) in the scientific literature despite the fact that juvenile salmon numerically dominate the inshore and coastal waters of Southeast Alaska [16] and some species of juvenile salmon have been found in schools or aggregations [17]. A review of the scientific literature revealed a single reference for salmon as prey for humpback whales [18]. Klumov [18] found adult pink salmon (*Oncorhynchus gorbuscha*) in the stomachs of humpback whales feeding near a run in the Kuril Islands of Russia.

Despite the lack of scientific record, hatchery personnel observed humpback whales feeding on juvenile salmon along shore near a release site as early as 1999. In 2008, a humpback whale was video recorded at Hidden Falls hatchery (electronic supplementary material, S1). In recent years (2011, 2015, 2016) of historically poor returns of chum salmon, hatchery managers have implicated humpback whale predation. Modified rearing and release protocols have been implemented to minimize humpback whale predation [19–21], but the success of these strategies is difficult to measure.

The objectives of this study were to document juvenile salmon as prey for humpback whales in Southeast Alaska, model the main factors affecting the probability of sighting humpback whales at release sites and describe humpback whale foraging behaviours at these sites.

## 2. Material and methods

### 2.1. Study area

**Figure 1.**
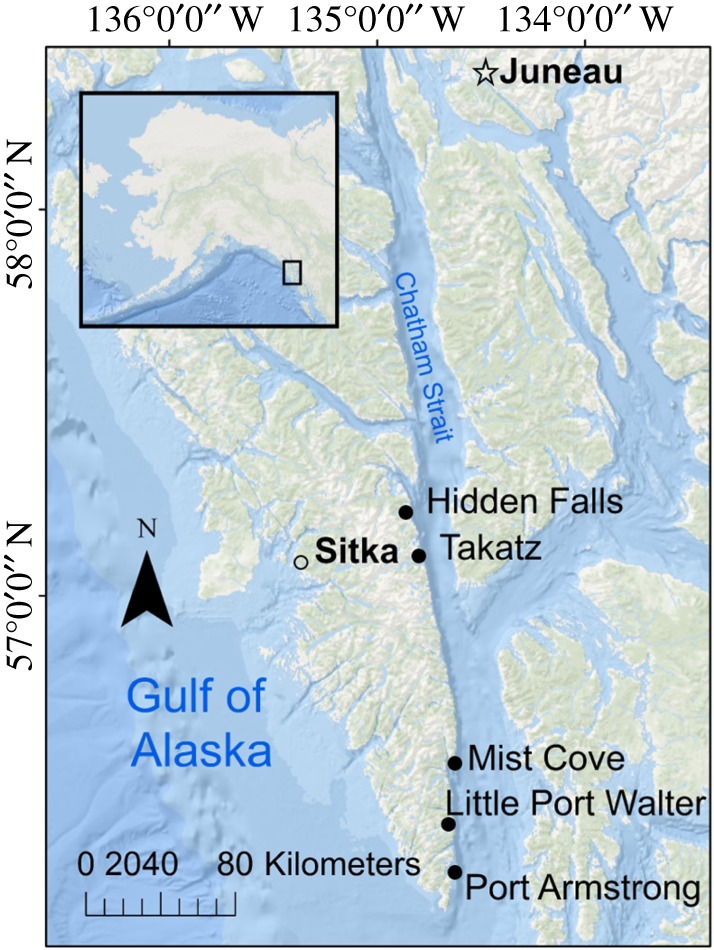
Participating hatchery release sites on Eastern Baranof Island along Chatham Strait. Release sites are shown with dark dots. Cities Juneau and Sitka are shown for reference.

This study was located at five hatchery release sites in protected coves on the eastern side of Baranof Island in Southeast Alaska, adjacent to Chatham Strait, a deep (up to 600 m), 240 km long, 15 km wide channel within the Alexander Archipelago (figure 1). Three different organizations participated in data collection at five release sites: Hidden Falls, managed by the Northern Southeast Regional Aquaculture Association (NSRAA); Takatz (NSRAA); Mist Cove (NSRAA); Little Port Walter (NOAA) and Port Armstrong (Armstrong Keta Inc.).

### 2.2. Hatchery processes

Salmon hatcheries have operated sporadically in Southeast Alaska since the late nineteenth century [22]. In the 1970s, production increased to augment low wild stock catches and abundance. Releases increased until the mid-1990s with over 400 million juvenile salmon released annually in Southeast Alaska [23,24]. In Alaska, salmon hatcheries fertilize eggs and rear hatchlings in captivity. After 6 to 18 months, salmon are transferred to floating saltwater net pens for acclimatization prior to ocean release [23]. Specific rearing practices vary by site and species, with longer rearing times generally leading to fewer, larger fish at the time of release. Chinook (*O. tshawytscha*) and coho salmon (*O. kisutch*) are typically released at larger sizes than pink and chum salmon (*O. keta*). After release, salmon are not restrained or fed and must eventually make their way to the open ocean, comingling with wild salmon. The salmon that survive to adulthood are then caught by commercial, sport and personal-use fisheries as they make their way back to the release sites to spawn [25]. The five sites included in our study release Chinook, coho, chum and pink salmon. The numbers and species released vary substantially by site and, to a lesser extent, by year. Hidden Falls releases Chinook, coho and chum salmon; Takatz releases chum salmon only; Mist Cove releases coho salmon only; Little Port Walter releases Chinook salmon only; and Port Armstrong releases all four species. Hidden Falls and Port Armstrong released the largest biomasses annually (mean of 190 000 and 160 000 kg, respectively) followed by Takatz (110 000 kg), Mist Cove (47 000 kg) and Little Port Walter (3500 kg) (appendix A). Chum salmon are the species released in greatest abundance and with the greatest economic importance in this region [26].

### 2.3. Behavioural observations at release sites

A standardized data collection protocol was developed in collaboration with hatchery managers. Each organization designated observers from among their on-site staff to participate in behavioural data collection. Behavioural observations were conducted at each of the five release sites over 6 years (2010–2015). Each site was systematically sampled twice a day, once in the morning and once in the afternoon. Observation times were selected by observers at the beginning of each season and remained consistent throughout the season to prevent biasing observations towards low-probability events. Whale observations outside of the predetermined sampling times were designated separately as opportunistic sightings. Observations were to begin about a week prior to releases and conclude one to two weeks after releases, when possible. Observations were recorded on standardized forms, with information on humpback whale presence, abundance and behaviours (sleeping/logging, breaching, surface feeding), as well as the presence of other possible salmon predators. Forms included a list of physical barriers whales may have used to aid in prey capture. This list was modified from a list of barriers compiled by the Glacier Bay Humpback Whale Monitoring Programme (C. Gabriele 2017, personal communication) to include net pens and docks in addition to surface, shoreline, tide-rip and kelp. Observers also noted the timing, location, species, abundance, age and mass of juvenile salmon released. During the coho salmon release at Hidden Falls in 2014, photography and videography were used to document feeding events (Go Pro Hero 4).

To determine which factors affected the presence of humpback whales at release sites, we modelled the probability of sighting a whale at a hatchery release site using a logistic generalized linear model (GLM) and Akaike Information Criteria (AICc)-based model selection [27,28]. Tested covariates included release site, year and a categorical variable for timing of the observation period relative to releases. Staff occasionally extended their observations beyond 15 min, which could increase the probability of sighting a whale. We, therefore, included observation duration as a covariate, and all model results were presented based on model predictions for a 15-min observation period:

2.1 $$\ln\;\left(\frac{\pi_{ijk}}{1-\pi_{ijk}}\right)=\beta_{0}+\beta_{1}({\mathrm{year}}_{i})+{\mathrm{site}}_{j}\times {\mathrm{timing}}_{k}+\beta_{2}({\mathrm{duration}}_{i})+e_{i},$$

where *π_ijk_* is the probability of observing a whale in year *i* at site *j* with timing *k*; year, an ordinal variable from 2010 to 2015; site, a categorical variable with five factor levels (*j*) for the five release sites (Hidden Falls, Takatz, Mist Cove, Little Port Walter and Port Armstrong); timing, a categorical variable referring to the timing of the observation with reference to the release season defined by the first release of the year and the last release of the year from that site with three factor levels (*k*): before, during and after; and duration, a continuous variable describing the total duration of observation effort expressed as a fraction of 24 h.

For observations conducted after the final release, an additional covariate (f.release) for the number of elapsed days since the last release was included.

For *k = *after

2.2 $$\ln\;\left(\frac{\pi_{j(k=\mathrm{after})}}{1-\pi_{j(k=\mathrm{after})}}\right)=\beta_{0}+\beta_{1}(\mathrm{year})+{\mathrm{site}}_{j}+\beta_{2}(\mathrm{duration})+\beta_{3}(f.\mathrm{release})+e_{i},$$

where *π_j_*_(*k=*after)_ is the probability of observing a whale in year *i* at site *j* after the final release at that site has occurred (i.e. *k* = after) where other variables are defined identically to the above and f.release is an ordinal variable that expresses the number of days that have elapsed since the final release at a particular site in a particular year.

## 3. Results

### 3.1. Humpback whale feeding behaviour at release sites

**Figure 2.**
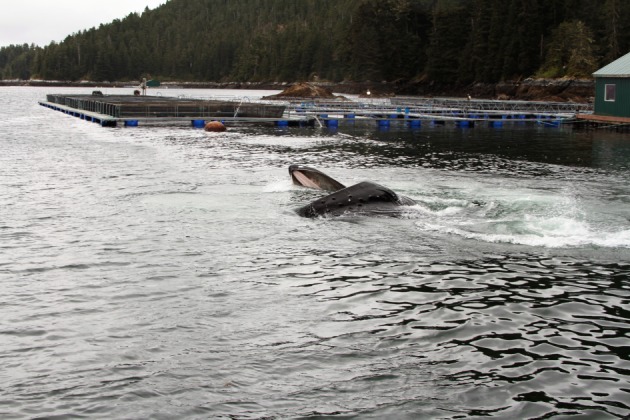
Humpback whales feeding in front of saltwater holding pens for salmon after a release in May 2014.

Observers recorded data on the presence or absence of humpback whales during 2252 observation periods at five hatchery release sites over 6 years. Humpback whales were reported to be targeting releases of Chinook, chum and coho salmon. For each of these three species, whales were observed feeding when no other species had been released from that site. Underwater video and photographs showed humpback whales targeting coho salmon at Hidden Falls hatchery in 2014 (figure 2; electronic supplementary material, S2). When humpback whales were noted near the release sites (*n* = 124 sightings), 81% of those sightings were of single individuals (*n* = 100); 10% of whale observations had a group size of 2 (*n* = 13) and 9% were 3 or more whales (*n* = 11) with a single observation of 10 animals, although this group was specifically noted as feeding on herring (*Clupea pallasii*). For 60% of observations when whales were sighted (*n* = 75), at least one barrier was noted. For the remaining 40% of whale sightings (*n* = 49), observers did not note feeding in the presence of a feeding barrier, noted that no feeding barrier was present or were uncertain. The most common barriers noted other than the surface (presumed for any observed feeding events) were shoreline (42%), bubbles (27%), dock or net pen (16%), tide (5%) and kelp (2%). Multiple feeding barriers were recorded in 26% of observations. In addition to these feeding behaviours, whales were noted as sleeping/logging (i.e. holding stationary at the surface; 2%) and breaching (5%).

### 3.2. Probability of sighting a whale

**Figure 3.**
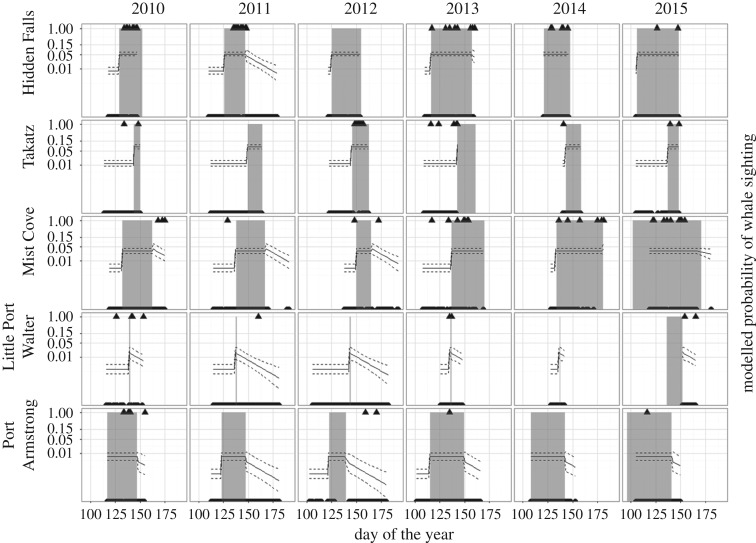
Probability (solid line) of sighting a whale before, during (grey shading) or after the release period at five hatcheries. The triangles on the top of each panel represent whale sightings, on the bottom represent observations where no whales were observed. Probabilities were generated from the top-ranked binomial models from tables 1 and 2. Dashed lines represent standard errors.

When modelling the probability of whale sightings over all time periods, the best models included site, timing and observation duration as explanatory variables (table 1). The probability of whale sightings increased notably once salmon were released (figure 3). Probability of whale sightings was highest at the Takatz and Hidden Falls sites, and lowest at Port Armstrong. As expected, the probability of a whale sighting increased with observation duration. Overall, the probability of whale sightings decreased with year, but year was not included in the top-ranked model (including year resulted in ΔAICc = 0.6 from the top model).

**Table 1.** Top candidate logistic models for describing the probability of sighting a humpback whale at a release site (response) based on temporal and spatial predictors. All models include an intercept term. The full model (EQ1) is shown here as the third-ranked model. *K* is the total number of parameters, AICc is the Akaike Information Criterion adjusted for sample size. ΔAICc is the difference between the AICc for each model and the AICc for the top-ranked model.

rank	model parameters	*K*	residual dev.	AICc	ΔAICc
1	site + timing + duration	9	844.80	860.9	0.0
2	site + timing + duration + year	10	843.44	861.5	0.6
3	site + timing + duration + site : timing	16	834.46	864.7	3.8
4	site + timing + duration + year + site : timing	17	833.04	865.3	4.4
5	site + timing + year	9	870.90	887.0	26.1
6	site + timing	8	874.38	888.4	27.6

**Table 2.** Top candidate logistic models for describing the probability of sighting a humpback whale at a release site (response) after releases have concluded. All models include an intercept term. The full model (EQ2) is shown here as the second-ranked model. *K* is the total number of parameters, AICc is the Akaike Information Criterion adjusted for sample size. ΔAICc is the difference between the AICc for each model and the AICc for the top-ranked model.

rank	model parameters	*K*	residual dev.	AICc	ΔAICc
1	duration + site + f.release	7	138.1	150.3	0.0
2	duration + site + f.release + year	8	137.1	151.3	1.0
3	duration + site	6	143.5	153.6	3.3
4	duration + site + year	7	141.6	153.8	3.5
5	duration + f.release	4	148.7	154.8	4.5
6	duration + f.release + year	5	148.7	156.8	6.5

At several hatcheries, there was considerable variability in the frequency of whale observations among years (figure 3). For example, Hidden Falls recorded no whales observed during scheduled observation periods in 2012 despite frequent observations in 2010 and 2013; however, whales were not entirely absent, as they were noted opportunistically. The following predicted probabilities for 15 min observation periods during the release season at each site were generated from the top-ranked model: Takatz (0.08) and Hidden Falls (0.05) compared with Mist Cove (0.03), Little Port Walter (0.02) and Port Armstrong (0.01).

For the final-release model (table 2), we found again that site and observation duration (coefficient again positive) were important predictors. Also important was elapsed time since the final release (f.release). These variables were included in the top two models and its coefficient was negative in all tested models, causing predicted probabilities to decrease with time after the final release. As in the overall model, year was less important and again excluded from the top model (ΔAICc = 1).

## 4. Discussion

Here we document humpback whales feeding on a novel prey. These feeding events were documented with direct observations as well as photographic and videographic evidence from the hatchery release sites. Using standardized observation methodology, we determined that humpback whale presence was closely associated with the release of juvenile salmon. Hatchery-released salmon were abundant in the region for only a few decades [23] before whales began to exploit them annually at multiple sites. The rapid release of large numbers of juvenile hatchery salmon, which differs from the protracted marine migration of their wild conspecifics, probably increased their profitability as prey for humpback whales, which rely on dense aggregations of prey. Wild Chinook and coho salmon in particular are known for agonistic behaviours and diffuse distributions [29,30], which may make them atypical prey for filter-feeding whales in a natural system. The extent to which humpback whales may target wild salmon or hatchery-released salmon after their outmigration from the release sites is unknown.

As expected, humpback whales were most common while releases were in progress; however, whales were also seen prior to and following releases. It is possible that pre-release humpback whale observations reflect whales assessing the prey field periodically in anticipation of a release but only spending time there when sufficient prey are encountered. Prey anticipation by Dolly Varden (*Salvelinus malma*) has been noted at release sites in Southeast Alaska (E. Prestegard 2016, personal communication) and by sculpin (*Cottus* spp.) anticipating spawning sockeye salmon (*O. nerka*) [31]. The decline in whale sightings after releases have concluded is best explained by a decrease in prey availability due to the dispersal and mortality of juvenile salmon from the release area. These local declines in humpback whale sightings were notable because they occur despite a concomitant seasonal increase in humpback whale populations in the region [15].

Hidden Falls and Takatz had the highest rates of whale sightings. Hidden Falls and Port Armstrong release the greatest biomass of salmon each year, but Takatz and Hidden Falls are located near each other and are not truly independent, with whales and potentially also salmon moving between these areas. Hidden Falls, Takatz and Mist Cove also tend to release salmon later than more southerly sites. The later timing of these releases (May and June) compared to Port Armstrong (April and May) may correspond with more whales present on the feeding grounds following their spring migration [15].

While at release sites, humpback whales often fed near physical barriers. Whales feeding near barriers may simply be a result of salmon distribution near these structures, or conversely feeding near barriers could be a tactic used by whales for aggregating prey or impeding prey escape. The frequent use of bubbles offered stronger evidence of forced prey aggregation. Feeding near barriers has been observed and noted by Glacier Bay National Park researchers for decades (Glacier Bay 2017, unpublished data). It has also been offered as an explanation for the use of bubbles to corral prey as well as near shore, tidally mediated and surface-feeding behaviours [32–34]. These behaviours may be necessary to aggregate Chinook and coho salmon into a sufficient density for profitable feeding, as these species do not school as densely as pink, chum or sockeye salmon juveniles [17,35]. Species preference could not be directly tested because at Hidden Falls and Port Armstrong, multiple species were released in succession, with overlapping presence at the release site. In addition, species releases at single-species sites were confounded by differences in biomass, release timing and location.

Despite the increase in humpback whales regionally and the relatively recent introduction of hatchery salmon as a prey source, we found no evidence of an increasing trend in humpback whale predation at release sites across years. One explanation is that the resource is currently being fully exploited at these release sites and the prey or habitat characteristics cannot support more frequent feeding. It is also possible that hatcheries are not particularly favourable places to feed compared to other foraging opportunities available to humpback whales. This is supported by the observation of whales feeding predominantly as individuals rather than feeding aggregations. Finally, it may be too soon to detect an increase over the substantial interannual variability. Even if these sites are fully exploited, hatchery predation could still be spreading to other releases sites in the region. If recent increases in the humpback whale population both locally and throughout the North Pacific [13,14] result in increased intraspecific competition, one possible outcome is increased dietary diversity of the population via individual specialization on less-preferred prey [36].

The interaction between humpback whales and an anthropogenically derived food source bears further investigation as both a novel predator–prey interaction and for the potential economic impact. Future studies will directly test whether high humpback whale predation on a salmon cohort at the point of release is related to poor marine survival of released salmon and assess the economic impacts of that predation to local fisheries. During this study, hatchery staff noted many strategies for mitigating predation, primarily aimed at reducing the density of salmon aggregations at the releases site. One of the most widespread methods was to release fish slowly over time, a strategy known as a ‘trickle’ release as opposed to a more traditional ‘mass’ release. Staff also tried releasing fish at night, on an outgoing tide, or in a less sheltered location. The most intensive strategy employed by NSRAA was to release salmon at a larger size so that they will move from the littoral habitat more quickly [21]. A longitudinal study in space and time will be necessary to isolate the effects of these strategies on marine survival. Future studies will also characterize the prey field at release sites to determine the prey quality associated with foraging at hatchery release sites.

Phenotypic plasticity in foraging behaviour offers advantages over strict specialization under certain conditions [8]. Phenotypic plasticity that leads to dietary diversity across time, space or among individuals can be an important evolutionary strategy to persist or thrive in changing environmental conditions [7] or high intraspecific competition [9]. The resulting behavioural innovations may be a key reason why humpback whale populations have recovered so successfully in much of the world [37] and Southeast Alaska, in particular [14].

## Data accessibility

All data are available through the Dryad Digital Repository: http://dx.doi.org/10.5061/dryad.ms75s [38].

## Appendix A. Release data for five hatcheries over 6 years.

**Table:**

hatchery	year	species	*n* released	mean mass (g)	start of releases	end releases
Hidden Falls	2010	Chinook	940 000	69.8	28 May 2010	1 June 2010
		chum	40 268 000	2.1	13 May 2010	22 May 2010
		coho	2 060 000	21.3	9 May 2010	26 May 2010
	2011	Chinook	535 000	53.1	13 May 2011	18 May 2011
		chum	37 601 000	2.2	20 May 2011	27 May 2011
		coho	3 048 000	21.7	6 May 2011	27 May 2011
	2012	Chinook	523 000	55.1	7 May 2012	10 May 2012
		chum	46 246 000	2.3	18 May 2012	2 June 2012
		coho	2 209 000	21.9	4 May 2012	26 May 2012
	2013	Chinook	518 000	61.8	26 April 2013	8 May 2013
		chum	34 867 000	2.6	17 May 2013	3 June 2013
		coho	3 137 000	23.5	1 May 2013	6 June 2013
	2014	Chinook	558 000	66.8	1 May 2014	4 May 2014
		chum	26 035 000	2.2	21 May 2014	7 June 2014
		coho	2 685 000	24.2	5 May 2014	27 May 2014
	2015	Chinook	736 947	64.6	16 April 2015	14 May 2015
		chum	28 416 000	2.6	12 May 2015	28 May 2015
		coho	3 235 598	24.2	3 May 2015	28 May 2015
Takatz	2010	chum	39 039 000	2.1	24 May 2010	30 May 2010
	2011		38 901 000	2.5	29 May 2011	13 June 2011
	2012		40 447 000	2.5	24 May 2012	10 June 2012
	2013		39 654 000	2.8	23 May 2013	10 June 2013
	2014		42 433 000	2.8	23 May 2014	7 June 2014
	2015		43 224 000	2.8	17 May 2015	28 May 2015
Mist Cove	2010	coho	1 193 000	16.4	12 May 2010	11 June 2010
	2011		647 000	22.3	17 May 2011	16 June 2011
	2012		2 015 000	19.3	29 May 2012	13 June 2012
	2013		2 567 000	20.8	17 May 2013	19 June 2013
	2014		2 417 000	23.8	13 May 2014	29 June 2014
	2015		2 498 000	39.7	12 April 2015	20 June 2015
Little Port Walter	2010	Chinook	238 000	18.1	19 May 2010	19 May 2010
	2011		180 000	22.0	17 May 2011	17 May 2011
	2012		150 000	16.2	22 May 2012	22 May 2012
	2013		139 000	16.9	15 May 2013	16 May 2013
	2014		211 000	28.5	16 May 2014	16 May 2014
	2015		149 000	14.7	16 May 2015	1 June 2015
Port Armstrong	2010	Chinook	276 000	31.7	8 May 2010	17 May 2010
		chum	27 296 000	1.2	27 April 2010	27 April 2010
		coho	3 224 000	17.1	8 May 2010	27 May 2010
		pink	53 677 000	0.5	29 April 2010	29 April 2010
	2011	Chinook	250 000	30.0	15 May 2011	15 May 2011
		chum	28 445 000	1.3	7 May 2011	7 May 2011
		coho	1 757 000	18.5	15 May 2011	27 May 2011
		pink	75 506 000	0.5	3 May 2011	7 May 2011
	2012	Chinook	402 000	41.9	12 May 2012	18 May 2012
		chum	52 919 000	1.9	1 May 2012	1 May 2012
		coho	4 761 000	19.7	18 May 2012	18 May 2012
		pink	82 734 000	0.5	1 May 2012	1 May 2012
	2013	Chinook	239 000	13.6	14 May 2013	14 May 2013
		chum	31 525 000	1.8	25 April 2013	4 May 2013
		coho	2 462 000	25.7	18 May 2013	29 May 2013
		pink	52 090 000	0.7	25 April 2013	4 May 2013
	2014	Chinook	161 000	14.7	14 May 2014	14 May 2014
		chum	25 029 000	2.4	25 April 2014	30 April 2014
		coho	1 748 000	24.3	17 May 2014	22 May 2014
		pink	79 659 000	0.4	18 April 2014	7 May 2014
	2015	Chinook	508 000	21.0	8 May 2015	17 May 2015
		chum	22 817 000	3.0	6 April 2015	11 April 2015
		coho	1 945 000	25.3	15 May 2015	21 May 2015
		pink	87 665 000	0.7	20 April 2015	6 May 2015
